# Relationship Between COVID-19 Infection and Liver Injury: A Review of Recent Data

**DOI:** 10.3389/fmed.2020.00458

**Published:** 2020-07-21

**Authors:** Nurshad Ali

**Affiliations:** Department of Biochemistry and Molecular Biology, Shahjalal University of Science and Technology, Sylhet, Bangladesh

**Keywords:** SARS-CoV-2, COVID-19, liver enzymes, liver disease, liver injury

## Abstract

The COVID-19 outbreak is a great threat to public health worldwide. Lung injury is the main outcome of COVID-19 infection; however, damage can occur in other organs including the liver. Currently, limited data are available that link underlying liver injury with the severe SARS-CoV-2 infection. This review summarizes the available data on liver test abnormalities in COVID-19 patients; critically evaluates the possible causes of liver injury and provides recommendations for clinicians. In laboratory tests, serum levels of liver test markers notably transaminase, gamma-glutamyl transferase and total bilirubin were significantly higher in severe patients with COVID-19 infection. The use of certain drugs especially lopinavir and ritonavir showed an association with the progression of liver damage in severe cases. Available data suggest that liver injury in COVID-19 patients may result from direct effect by the virus, immune-mediated inflammation or drug-induced toxicity. Some studies demonstrated that COVID-19 patients with pre-existing liver disease are at higher risk for hospitalizations and mortality. Therefore, the impact of pre-existing liver disease on treatment and clinical outcomes of COVID-19 should be determined. Large-scale clinical studies are needed to identify the causes of liver injury in patients with COVID-19 infection.

## Introduction

The COVID-19 pandemic poses a great challenge to the international healthcare system. Older adults and those at any age with hypertension, coronary heart disease and diabetes are at higher risk of SARS-CoV-2 infection and severe disease course (1, 2). Although the lung is the main target organ of SARS-CoV-2 infection; damage can occur in multiple organs. The liver is the vital organ in the human body and its exposure to the viral particles might be an additional concern for CVID-19 patients. Up to now, there is no strong evidence that the liver cells are exposed to SARS-CoV-2 in severe cases. Moreover, it remains unclear yet to what extent liver diseases are considerable risk factors of COVID-19 severity and mortality.

Liver impairment is an emerging concern with COVID-19, as it was observed with the similar coronavirus SARS. According to previous studies, up to 60% of patients had a liver impairment, with liver biopsy specimens suggesting viral nucleic acid and damage (3–5). In these studies authors noted that this might have been the result of drug-induced liver damage, given that the majority of the patients were treated with high doses of antibiotics, hepatotoxic antiviral drugs and steroids.

Some studies have reported the clinical features and laboratory test results associated with liver dysfunction in patients with COVID-19 infection (6–13). Although, pre-existing liver conditions have not been listed in most of these studies and the interaction of pre-existing liver disease with COVID-19 has not been investigated, which are major limitations in evaluating the underlying causes of liver injury in the severe disease course. However, the elevated levels of alanine aminotransferase and reduced platelet counts and reduced levels of albumin showed an association with higher fatality in COVID-19 patients (2). It is still unknown whether these laboratory analyses are an indicator of pre-existing hepatic diseases in severe patients, whether they rather mirror liver failure caused by the SARS-CoV-2 itself (14). Overreaction of the immune system may also contribute to disease progression and can lead to liver injury (15). Serum concentrations of pro-inflammatory cytokines, including C-reactive protein (CRP) TNF-α, IL-1β, and IL-6 were seen at elevated levels in most of the severe cases, indicating cytokine storm syndrome might be associated with the disease severity (16, 17). It is also considered that systemic infections might influence liver functions in a severe disease course. Further clinical studies can provide a better insight on liver damage in severe patients with SARS-CoV-2 infection. This review summarizes the available data on liver test abnormalities in COVID-19 patients; critically evaluates the possible causes of liver injury and provides recommendations for clinicians. Online databases including Google Scholar, PubMed, Scopus, and medRxiv were searched to identify relevant publications using relevant keywords.

## Prevalence of Liver Function Test Abnormalities in COVID-19 Patients

Several studies have demonstrated the different degrees of elevated liver test markers in COVID-19 patients, mainly reported by alanine aminotransferase (ALT), aspartate aminotransferase (AST), gamma-glutamyl transferase (GGT) levels accompanied by moderately elevated prothrombin time (PT), and total bilirubin (TB) levels (6–13, 17–27). However, there are limited numbers of studies that have specifically investigated the clinical characteristics of liver failure in COVID-19 patients. The available reports on liver function tests in COVID-19 patients are listed in Table 1. Of these studies, an increasing trend for liver enzymes has been observed in severe/non-survivor patients. The prevalence of elevated ALT, AST, and GGT were up to 82, 75, and 72%, respectively. A recent study reported 76.3% of liver test abnormalities and 21.5% of liver injury among hospitalized COVID-19 patients (6). Serum levels of ALT, AST, GGT and TB were significantly higher (*p* < 0.01) in ICU admitted severe patients than non-severe patients (6). In that study, 26.7% of patients with liver test abnormalities showed a progression to severe pneumonia. The abnormal liver tests were more pronounced in patients within 2 weeks of hospitalization. The liver injury was significantly higher in severe patients than non-severe patients. The authors also noted that patients in hepatocyte type had significantly higher odds for progression to severe COVID-19. In another study, concentrations of ALT, AST, GGT, ALP, and TB were markedly higher in deceased patients than in recovered patients (19). About 52% of deceased patients and 16% who recovered had elevated AST levels (19). In a retrospective study, the levels of ALT, AST, GGT, and TB, showed statistically significant elevation in severe COVID-19 patients compared with that in mild patients (27). ALT and AST abnormalities were also found at least two times higher in severe patients than non-severe patients (13). Moreover, a large cohort study including 1,099 patients, reported an elevated level of ALT and AST (28.1 and 39.4%, respectively) in severe patients than non-severe patients (19.8 and 18.2%, respectively) (8). Similarly, the prevalence of elevated AST in ICU patients (62%) was higher than non-ICU patients (25%) (28). Furthermore, in another publication, AST abnormalities were found lower in patients before the onset of symptoms than the patients did diagnose after the onset of symptoms (9). Other studies, however, reported different results. For example, Wu et al. did not find significant differences in liver function tests when compared to mild cases with severe cases (26). Another study consisted of 339 elderly COVID-19 patients and reported no evident differences in ALT levels between death and survival (25). Therefore, researchers also argue that though liver function tests abnormalities are common in COVID-19 patients, liver functions impairment maybe not a prominent feature of COVID-19 (27). However, the major studies clearly indicated increased liver dysfunctions in severe COVID-19 patients, though clinically significant liver failure has rarely been described.

**Table 1:** Liver function test abnormalities in COVID-19 patients.

**References**	**Group**	**Patients (*n*)**	**ALT (U/L)**	**AST (U/L)**	**GGT (U/L)**	**ALP (U/L)**	**Prothrombin time (s)**	**Total bilirubin (μmol/L)**	**% of patient with abnormal-liver tests**
Cai et al. (6)	Severe	85	67^b^ (47–100)	58^b^ (41–93)	92^c^ (53–161)	79 (62–101)	NA	22^c^ (18–28)	ALT: 82.3, AST: 75.3 GGT: 72.3, ALP: 12.2
	Non-severe	233	41 (23–65)	34 (27–45)	40 (25–61)	69 (57–89)	NA	19 (13–26)	ALT: 52.2, AST: 26.9 GGT: 39.1, ALP: 10.5
Cai et al. (18)	Severe	58	42^c^ (28–72)	38^c^ (23–65.5)	47^c^ (31–152)	87 (54–106)	NA	20.7^c^ (12.7–25.3)	ALT: 34.5, AST: 24.1 GGT: 43.1, ALP: 1.7
	Non-severe	240	22 (22–38)	28 (19–37)	33.6 (22.3–43.5)	82 (49–98)	NA	13.5 (8.1–17.5)	ALT: 7.9, AST: 4.6 GGT: 10.8
Chen et al. (19)	Deaths	113	28 (18–47)	45 (31–67)	42 (27–70)	76 (60–118)	15.5 (14.4–17.3)	12.6 (9.4–16.2)	ALT: 27, AST: 52
	Recovered	161	20 (14.8–32)	25 (20–33.3)	28 (19–45.3)	64 (51–77)	13.9 (13.2–14.4)	8.4 (5.8–11.2)	ALT: 19, AST: 16
Chen et al. (7)	Hospitalized	99	39 (22–53)	34 (26–48)	NA	NA	11.3 (1.9)	15.1 (7.3)	ALT: 28,.AST: 35
Du et al. (20)	Fatal cases	85	72.9 (199.5)	94.4 (263.3)	NA	NA	15.4 (3.3)	18.4 (13.6)	ALT: 16.5, AST: 32.9
Guan et al. (8)	Severe	173	NA	NA	NA	NA	NA	NA	ALT: 28.1, AST: 39.4
	Non–sever	926	NA	NA	NA	NA	NA	NA	ALT: 19.8, AST: 18.2
Huang et al. (28)	Severe	13	49^a^ (29–115)	44 (30–70)	NA	NA	12.2^a^ (11.2–13.4)	14^a^ (11.9–32.9)	AST: 62
	Non-severe	28	27 (19.5–40)	34 (24–40.5)	NA	NA	10.7 (9.8–12.1)	10.8 (9.4–12.3)	AST: 25
Huang et al. (21)	Non-survivors	36	26 (18–38)	43 (30–51)	NA	NA	NA	11.2 (7.5–19.2)	ALT:13.3, AST: 58.1
Jin et al. (22)	Severe	74	25 (15.7–38.5)	29.4^a^ (20.8–38.6)	NA	NA	NA	10	NA
	Mild	577	21.5 (15–32.8)	24.4 (19–32)	NA	NA	NA	9.6	NA
Liu et al. (17)	Severe	13	27 (23–50)	51.2	NA	NA	13.4	13.2	NA
	Mild	27	19 (13.5–26)	25.9	NA	NA	13.1	8.8	NA
Mo et al. (23)	Severe	85	28 (17–42)	37 (25–56)	NA	NA	NA	NA	NA
	Mild	70	20 (15–33)	32 (23–38)	NA	NA	NA	NA	NA
Pan et al. (24)	Severe	103	42.2^a^	35.1^a^	NA	NA	NA	13.8	NA
	Mild	101	29.5	27.5	NA	NA	NA	13.5	NA
Shi et al. (9)	Hospitalized	81	46 (30)	41 (18)	NA	NA	10.7 (0.9)	11.9 (3.6)	53
Wang et al. (10)	Severe	36	35^b^ (19–57)	52^c^ (30–70)	NA	NA	13.2 (12.3–14.5)	11.5^a^ (9.6–18.6)	NA
	Non-severe	102	23 (15–36)	29 (21–38)	NA	NA	12.9 (12.3–13.4)	9.3 (8.2–12.8)	NA
Wang et al. (29)	Severe	16	23.9 (13.7–37.1)	NA	NA	NA	NA	15 (5.3–22.9)	NA
	Non-severe	193	19 (2.6–87.7)	NA	NA	NA	NA	10.9 (4–40.2)	NA
Wang et al. (25)	Deaths	65	24 (19–49)	43^c^ (30–68)	NA	NA	12.9 (11.9–14.1)	NA	NA
	Survivors	274	28 (17–43)	29 (22–43)	NA	NA	12 (11.6–12.6)	NA	NA
Wu et al. (26)	Severe	83	24 (18–38)	26 (23–39)	NA	NA	NA	6.7 (5.5–12.6)	NA
	Mild	197	20 (16–38)	26 (21–34)	NA	NA	NA	6.6 (5.2–12.1)	NA
Xu et al. (11)	Hospitalized	62	22 (14–34)	26 (20–32)	NA	NA	NA	NA	16.1
Yang et al. (12)	Survivors	20	NA	NA	NA	NA	10.9 (2.7)	13.1 (4.3)	30
	Non-survivors	32	NA	NA	NA	NA	12.9 (2.9)	19.5 (11.6)	28
Zhang et al. (27)	Severe	31	37.9^c^ (32.2)	38.9 ^c^ (22.6)	56.9^c^ (73.3)	79.5 (24.6)	NA	14.1^c^ (6.4)	ALT: 25.8, AST: 38.7 GGT: 16.1, ALP: 12.9
	Mild	84	21.2 (12.7)	24.4 (9.8)	28.5 (24.9)	71.6 (24.1)	NA	10.3 (4.3)	ALT: 3.6, AST: 5.9 GGT: 11.9, ALP: 2.4
Zhao et al. (13)	Severe	30	NA	NA	NA	NA	NA	NA	ALT: 16.7, AST: 26.7
	Mild	61	NA	NA	NA	NA	NA	NA	ALT: 8.2, AST: 16.4

*Data are presented as mean (SD) or median (IQR). Severe: patients admitted in the intensive care unit (ICU). NA, data was not available*.

*^a^P < 0.05, ^b^P < 0.01, ^c^P < 0.001 when mean or median value is compared to non-severe/mild cases*.

## Possible Effects of Drugs on Liver Function in Covid-19 Patients

It is also possible that the drugs used in the treatment are associated with liver injury in COVI19 patients. For example, the use of multiple drugs, such as antibiotics, antivirals, antipyretics and analgesics, and traditional Chinese medicine may cause liver injury in COVID-19 patients (30). Yet, there is no clear evidence that the liver dysfunctions during hospitalization are completely drug-induced in severe patients with COVI-19 infection. In a recent study, the liver biopsy specimen of a patient who died from COVID-19 showed elevated liver enzymes that could be the partial effects of drugs used in the treatment, and the liver dysfunction may be occurred because of sepsis and shock (6). It has been indicated that angiotensin II receptor blockers and ACE-inhibitors drugs may inhibit liver functions in COVID-19 patients (6). In that study, elevated levels of liver enzymes were observed among participants who used ACE-Is/ARBs drugs during hospitalization, though; the elevation was not significant with those who did not use these drugs (6). The authors also observed that the drugs lopinavir and ritonavir contributed significantly to liver test abnormalities and liver injury. These drugs increased the odds of liver injury by four-fold (*p* < 0.001). Moreover, using antibiotics in the treatment also showed an association with the increased prevalence of liver test abnormalities in the regression model; however in other models this association was not significant (6). Another study showed 55.4% of liver injuries after treatment with lopinavir and ritonavir, suggesting possible drug-induced liver damage in COVID-19 patients (30). A randomized controlled trial reported gastrointestinal adverse events in COVID-19 patients, who received lopinavir-ritonavir in the treatment (31). However, no remarkable differences were observed in the levels of liver test markers (ALT and AST) between the lopinavir-ritonavir group and standard care group of COVID-19 patients. Hydroxychloroquine, an antimalarial agent has not been associated with liver injury in COVID-19; however, it should be used with caution to avoid any harmful effects. Future studies would be worth conducting in determining the possible effects of drugs on liver function in COVID-19 patients.

## Possible Mechanisms of Liver Injury in Covid-19 Infection

It is speculated that liver injury in patients with SARS-CoV-2 infection may have occurred by directly the virus itself (32). Presence of diarrhea in 2–10% of patients, and SARS-CoV-2 RNA in blood and stools of patients with COVID-19 (33), indicating a possible viral inclusion in the liver (15). A recent review demonstrated that both SARS-CoV-2 and SARS-CoV use angiotensin-converting enzyme 2 (ACE2) receptor to enter the target host cell (10), which then subsequently infects the upper respiratory tract and lung cells. In pathological studies, patients with SARS showed the viral presence in their liver tissue at a low level (34), however, patients with MERS, virus were not detected in their liver tissue (35). Serum GGT, a potential diagnostic marker for cholangiocyte injury has been found at increased levels up to 72% in severe COVID-19 patients (6, 15). A preliminary study reported that the ACE2 receptor is abundantly expressed in cholangiocytes (36); suggesting that SARS-CoV-2 might bind to ACE2-positive cholangiocytes and cause liver dysfunction (15). Nonetheless, viral inclusions were not identified in the liver tissue of a patient who died from COVID-19 (37). Apart from these observations, it is also assumed that antiviral drugs used for the treatment might be linked with liver damage in COVID-19 patients (6, 15). Although, it remains unclear to what extent viruses or drugs are associated with liver injury in severe disease courses. Dysregulation of the innate immune response may be another important aspect of liver injury in COVID-19. So, the possible pathways that can be associated with liver damage in COVID-19 patients are (i) immune-mediated inflammation, such as cytokines storm and pneumonia-related hypoxia, (ii) Direct cytotoxicity because of active viral replication in the liver cells, (iii) Drug-induced liver damage: initially recommend antiviral drugs including lopinavir/ritonavir, chloroquine, remdesivir, tocilizumab, uminefovir, being potentially hepatotoxic in severe patients, (iv) Reactivation of pre-existing hepatic disease: patients with previous chronic hepatic disease are more vulnerable to hepatic damage from this viral infection, (v) Possible reactivation of hepatitis B virus with some biological drugs such as tocilizumab and baricitinib that may lead to liver dysfunction. Moreover, it is also unknown whether SARS -CoV−2 infection enhances cholestasis in patients with underlying cholestatic hepatic diseases. More mechanistic studies regarding virus entry and replication in liver cells and the potential consequences of drugs in the liver are required.

## Covid-19 Patients With Pre-Existing Liver Disease

Data on pre-existing liver disease in COVID-19 would be worth in evaluating the underlying causes of liver damage in severe disease courses. However, limited information is available on the interaction between pre-existing liver disease and COVID-19. A recent study examined the effects of pre-existing liver disease on outcomes in a large cohort of COVID-19 patients (*n* = 2,780) in the USA (38). The authors observed a higher proportion of comorbidities (diabetes 48% and hypertension 68%) in patients with liver disease. Fatty liver disease and non-alcoholic steatohepatitis (42%) were more frequent among patients with pre-existing liver disease. Importantly, the mortality rate was significantly higher (*p* < 0.001) in patients with pre-existing liver disease than patients without liver disease and the relative risk was markedly higher in patients with cirrhosis (p < 0.001) (38). Moreover, patients with pre-existing liver disease required increased hospitalization. Another recent study reported a high mortality rates from COVID-19 among patients with chronic liver disease and cirrhosis (39). The authors demonstrated that patients with liver cirrhosis could result in decompensation of the liver and mortality. Patients with cirrhosis had poor outcomes with an overall mortality rate of 40%. Those with decompensated cirrhosis had a mortality rate between 43 and 63%, compared with 12% for patients with hepatic disease but without cirrhosis (39). In that investigation, there were many COVID-19 positive patients who had no respiratory problems, suggesting that patients with chronic liver disease should get priority for COVID-19 testing even in the absence of typical COVID-19 symptoms. The probable reasons for poor outcomes in COVID-19 with pre-existing liver disease require further investigation; however, it seems that there is interplay between local liver injury and systemic disturbances (38).

## Management and Challenges of Liver Injury During Covid-19 Infection

Although, there is no direct evidence on liver failure in COVID-19 patients without pre-existing liver disease; several measures should be considered for all patients with COVID-19 during hospitalization. For example, individuals who are at high risk of hepatitis A infection should maintain personal hygiene and avoid large gatherings during this pandemic. Presently there is no evidence that people with hepatitis B and hepatitis C are at higher risk of infections. However, people living with hepatitis B and C should follow a healthy lifestyle and should also continue their regular treatments if they are affected by COVID-19 infection. People with pre-existing liver disease or after liver transplantation should follow the same preventive measures being followed by people with other medical conditions to avoid getting sick with or spreading COVID-19 infection. Moreover, attention should be paid to older people with severe medical conditions including liver test abnormalities as they are at higher risk of becoming severely ill from this infection. In addition, (i) the liver test markers should be measured and monitored regularly for all COVID-19 patients, (ii) Serologic testing for hepatitis B and C should take into consideration for COVID-19 patients during the measure of liver test biochemistries, (iii) Up to now, data are missing on the safety of drugs used for the treatment of COVID-19 patients with liver dysfunction, therefore, the detrimental effects of drugs on the liver injury during the treatment should be monitored and assessed frequently.

## Conclusions and Recommendations

Abnormal liver function test markers are more common in patients with severe COVID-infection. Available study findings support the hypothesis that liver injury might be associated with severe outcomes in COVID-19 patients. However, there is still lacks important data on larger studies to determine causality between COVID-19 and liver damage. The mechanism of liver injury in COVID-19 infection is not well-understood yet and seems to be multifactorial. The patients with advanced hepatic disease and those after liver transplantation are at high risk of progression to severe disease course. Therefore, it is recommended to identify and care for patients with chronic liver disease with priority within the limited healthcare resources. COVID-19 patients with liver injury are advised to be treated with drugs that are able to inhibit inflammatory responses and protect liver functions. In addition, the detrimental effects of certain drugs on the liver injury during hospitalization should be monitored and evaluated regularly. Attention should also pay toward modulating innate immune dysfunction in severe cases. Moreover, the effects of pre-existing liver disease on treatment and clinical outcomes of COVID-19 should be determined. Future research should focus on the potential causes of liver damage in severe patients with COVID-19 infection.

## Author Contributions

The author confirms being the sole contributor of this work and has approved it for publication.

## Conflict of Interest

The author declares that the research was conducted in the absence of any commercial or financial relationships that could be construed as a potential conflict of interest.
